# Neuronal Activity-Dependent Activation of Astroglial Calcineurin in Mouse Primary Hippocampal Cultures

**DOI:** 10.3390/ijms19102997

**Published:** 2018-09-30

**Authors:** Dmitry Lim, Lisa Mapelli, Pier Luigi Canonico, Francesco Moccia, Armando A. Genazzani

**Affiliations:** 1 Department of Pharmaceutical Sciences, Università del Piemonte Orientale “Amedeo Avogadro”, Via Bovio 6, 28100 Novara, Italy; pierluigi.canonico@uniupo.it (P.L.C.); armando.genazzani@uniupo.it (A.A.G.); 2 Department of Brain and Behavioral Sciences, University of Pavia, Via Forlanini 6, 27100 Pavia, Italy; lisa.mapelli@unipv.it; 3 Department of Biology and Biotechnology “Lazzaro Spallanzani”, University of Pavia, Via Forlanini 6, 27100 Pavia, Italy; francesco.moccia@unipv.it

**Keywords:** astrocytes, calcineurin, neuronal activity, NMDA, mGluR5, store-operated calcium entry

## Abstract

Astrocytes respond to neuronal activity by generating calcium signals which are implicated in the regulation of astroglial housekeeping functions and/or in modulation of synaptic transmission. We hypothesized that activity-induced calcium signals in astrocytes may activate calcineurin (CaN), a calcium/calmodulin-regulated protein phosphatase, implicated in neuropathology, but whose role in astroglial physiology remains unclear. We used a lentiviral vector expressing NFAT-EYFP (NY) fluorescent calcineurin sensor and a chemical protocol of LTP induction (cLTP) to show that, in mixed neuron-astrocytic hippocampal cultures, cLTP induced robust NY translocation into astrocyte nuclei and, hence, CaN activation. NY translocation was abolished by the CaN inhibitor FK506, and was not observed in pure astroglial cultures. Using Fura-2 single cell calcium imaging, we found sustained Ca^2+^ elevations in juxtaneuronal, but not distal, astrocytes. Pharmacological analysis revealed that both the Ca^2+^ signals and the nuclear NY translocation in astrocytes required NMDA and mGluR5 receptors and depended on extracellular Ca^2+^ entry via a store-operated mechanism. Our results provide a proof of principle that calcineurin in astrocytes may be activated in response to neuronal activity, thereby delineating a framework for investigating the role of astroglial CaN in the physiology of central nervous system.

## 1. Introduction

Astrocytes are an abundant non-neuronal cellular type in the brain [1]. They exert fundamental housekeeping and homeostatic functions in the central nervous system (CNS) and are also involved in the pathogenesis of many neurological diseases. Astrocytes are non-excitable cells as they are largely unable to generate action potentials in response to electrical or chemical stimulation. Conversely, astrocytes respond to extracellular stimuli by generating intracellular calcium signals by exploiting two main mechanisms: (i) activation of metabotropic receptors on the plasma membrane, leading to liberation of calcium ions from internal calcium stores; and (ii) a receptor/store-operated mechanism of calcium entry from the extracellular milieu through the plasma membrane [1]. Astroglial calcium signals are thought to have a number of implications for CNS pathophysiology, including modulation of synaptic release [2], synchronization of neuronal activity [3], regulation of frequency of spontaneous α-amino-3-hydroxy-5-methyl-4-isoxazolepropionic acid AMPA receptor currents [4] and participation in vesicular glutamate release [5]. Although the physiological role of calcium signals in astrocytes is still a matter of debate [6,7,8], there is broad consensus about their role and their alterations in brain pathology [9,10,11,12,13,14,15].

Calcineurin (CaN) is a calcium/calmodulin-activated serine-threonine phosphatase, which is highly expressed in the brain [16]. In neurons, CaN regulates neuronal excitability and synaptic transmission [17]. Moreover, CaN activation is associated with long-term depression [18], while CaN inactivation is required for establishment of aversive memory [19]. In astrocytes, calcineurin is principally involved in setting up reactive gliosis and neuroinflammation in a number of neuropathological conditions [20,21,22]. Conversely, activation of CaN in astrocytes has so far not been documented under physiological conditions and its role in housekeeping and homeostatic functions of astrocytes is currently unknown. 

The present work is designed as an in vitro proof of principle of astroglial CaN activation in response to neuronal activity. We show that long-term potentiation (LTP)-like neuronal activity robustly activates CaN in adjacent astrocytes and that store-operated Ca^2+^ entry through the astroglial plasma membrane is required for this process to occur.

## 2. Results

### 2.1. Neuronal Activity Leads to Activation of CaN in Astrocytes

**Figure 1:**
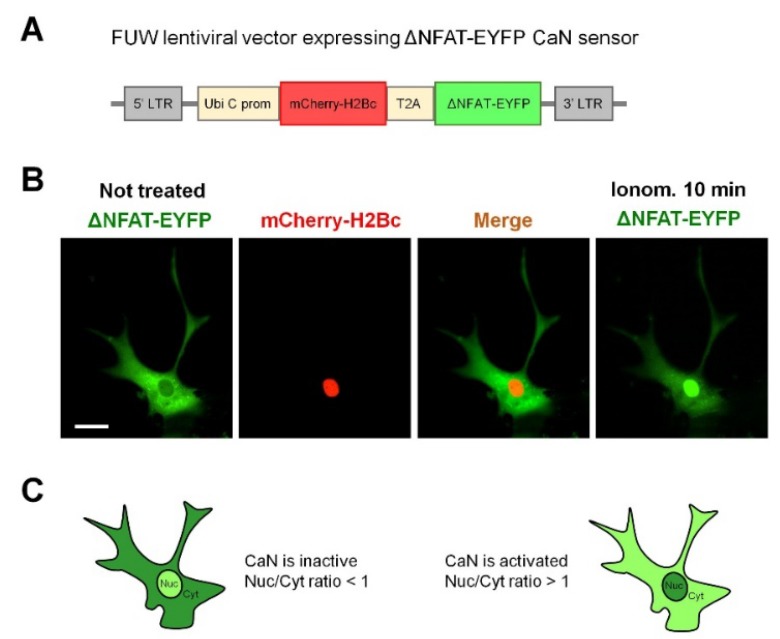
Validation of lentiviral vector expressing NY CaN sensor in astrocytes. (**A**) A scheme of lentiviral vector expressing ΔNFAT-EYFP (NY) CaN sensor. (**B**) Hippocampal astrocytes were transduced with NY CaN sensor and then stimulated with 2 μM ionomycin. Nuclear translocation of NY in astrocyte was observed after 10 min of incubation. A representative astrocyte is shown from three independent preparations. Bar, 10 μm. (**C**) Quantification of nuclear-to-cytosol ratio (Nuc/Cyt ratio) of NY in the green channel. CaN was considered inactive with Nuc/Cyt ratio <1, while Nuc/Cyt ratio >1 indicates CaN activation.

**Figure 2:**
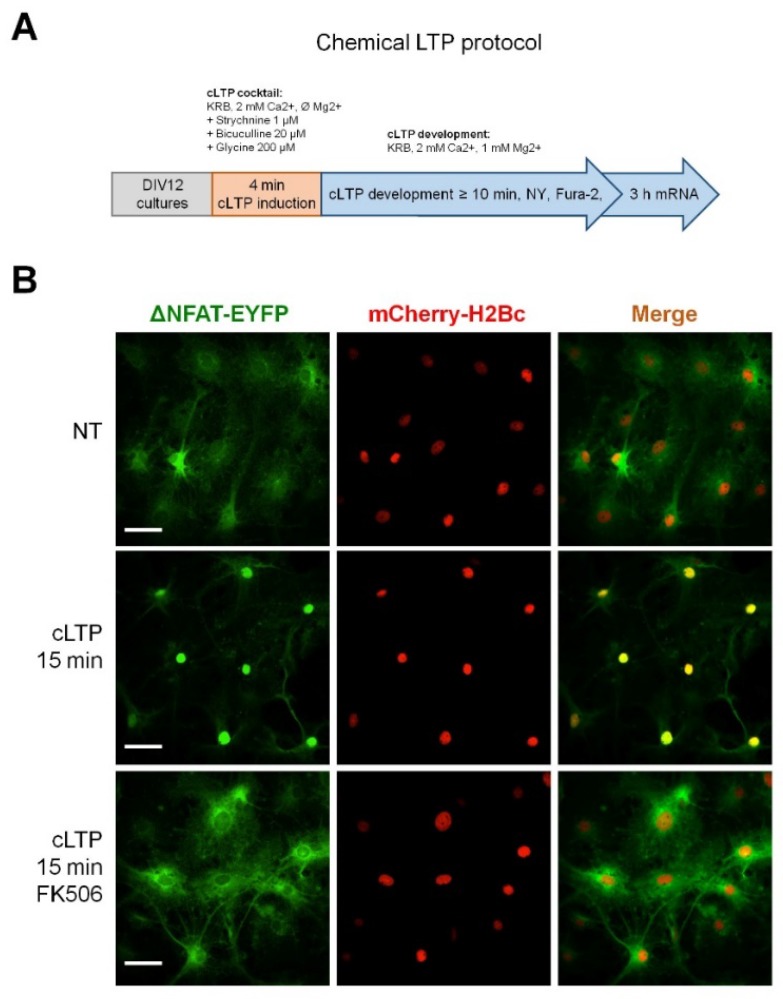
cLTP-induced NY nuclear translocation in astrocytes is CaN-dependent. (**A**) Scheme depicting cLTP induction protocol with consequent readouts. (**B**) Application of cLTP protocol to mixed neuron-astroglial hippocampal primary cultures induces robust NY translocation into the astrocyte nucleus at 15 min after cLTP induction phase. Note the complete inhibition of NY translocation when CaN inhibitor, FK506, was added during the cLTP development phase. Bar, 30 μm.

**Figure 3:**
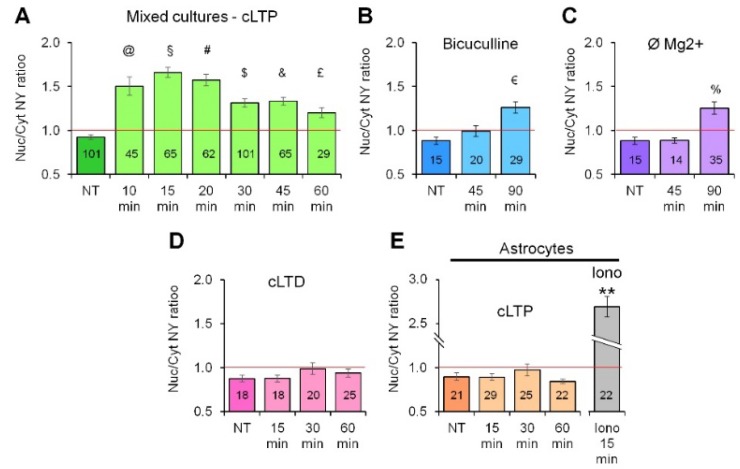
Quantification of NY Cyt/Nuc ratio after cLTP induction. (**A**) Time-course quantification of NY Cyt/Nuc ratio after cLTP induction in hippocampal mixed neuron-astroglial cultures. Note, nuclear localization of NY and hence, CaN activation, is evident already 10 min after cLTP induction, reaches maximum at 15–20 min and is still evident after 1 h of cLTP induction. Continuous bicuculline (40 μM) treatment (**B**) and Mg^2+^ withdrawal (**C**) are sufficient to induce NY translocation into astrocyte nucleus, although more than 1 h is required for the translocation to occur. Nuclear NY translocation in astrocytes was not observed upon application of chemical long-term depression protocol (cLTD) (**D**) or when purified astroglial cultures were stimulated with cLTP protocol (**E**). Ionomycin-induced (2 μM) NY translocation ratio is included as a positive control (**E**). Data are expressed as mean ± SEM of indicated number of cells measured, as indicated in materials and methods, from 2–3 coverslips (technical replicates) for each of three independent culture preparation (biological replicates). ^@^, *p* = 1.7 × 10^−8^; ^§^, *p* = 2.1 × 10^−18^; ^#^, *p* = 2.15 × 10^−14^; ^$^, *p* = 1.5 ×10^−8^; ^&^, *p* = 4.01 × 10^−8^; ^£^, *p* = 0.0058; ^€^, *p* = 0.00015; ^%^, *p* =0.00109; **, 1.3 × 10^−10^, all vs. NT.

Fluorescent CaN probe based on a transcription-deficient truncated variant of nuclear factor of activated T-cells c2 (NFATc2) fused with EYFP (NY) (Figure 1A), has been used in immune cells to monitor CaN activation in vivo [23]. To evaluate whether NY works in astrocytes, we have transduced an astroglial culture with NY-expressing lentiviral particles and monitored EYFP fluorescence in response to stimulation with ionomycin (1 μM), a Ca^2+^ ionophore widely used to activate CaN in different cellular types [24,25,26,27]. Figure 1B and Video S1 show that, after about 10 min of treatment, NY robustly translocates into the nucleus. We also checked the tropism of lentiviral particles pseudotyped with vesicular stomatitis virus protein G (VSVG) to neuronal and astroglial cells in culture. We found that astrocytes are efficiently transduced by the vector with occasional transduction of some neurons (<1%). Taking advantage of the preferential astrocytic transduction of the NY probe, we investigated whether chemical induction of neuronal activity was able to activate CaN in astrocytes. To achieve this goal, we have chosen a widely used protocol for chemical induction of long term potentiation (cLTP) in brain slices and in cultured neurons which consisted in short (4 min) application of a cocktail containing bicuculline (20 μM), strychnine (1 μM) and glycine (20 μM) in Mg^2+^-free Krebs-Ringer buffer (KRB) buffer supplemented with 2 mM Ca^2+^ (cLTP cocktail, the induction phase), followed by washout of the cLTP cocktail and addition of KRB containing 1 mM Mg^2+^ and 2 mM Ca^2+^ (KRB + Mg + CaMg, the expression phase, Figure 2A). We applied the cLTP protocol to mixed hippocampal astrocyte-neuronal cultures, expressing NY probe, at DIV12–14. At different time-points after stimulation, the cells were fixed and NY nuclear translocation was quantified by calculating nuclear-to-cytosol fluorescence ratio (Nuc/Cyt ratio, Figure 1C, see also Methods section for details). Co-expression of histone 2B-fused mCherry allowed reliable localization of nuclear NY even in astrocytes expressing low NY levels or superimposed with neurons. Already 10 min after cLTP induction, nuclear localization of NY was detected as judged by the increased Nuc/Cyt NY fluorescence ratio (Figure 3A). 15 min after cLTP induction, a robust nuclear NY translocation was observed (Figure 2B). The histogram in Figure 3A shows that elevated Nuc/Cyt NY ratio can be observed at least until 1 h after cLTP induction indicating that the protocol induced robust and long lasting CaN activation. This activation was completely abolished if either FK506 (200 nM; Figure 2B) or cyclosporine A (500 μM), two well-known CaN inhibitors, were added to KRB + Mg + Ca during cLTP development.

Alongside the cLTP protocol, we used other strategies to induce neuronal activity, namely bicuculline alone at 40 μM in Mg^2+^-free KRB (Figure 3B) and KRB with Ø Mg^2+^ without bicuculline (Figure 3C). Both protocols produced delayed (90 min of continuous treatment) translocation of NY, indicating that the increase in neuronal activity alongside the de-inactivation of *N*-methyl-d-aspartate receptors is sufficient to produce activation of CaN in astrocytes. Interestingly, NY nuclear translocation was not observed after application of a widely used protocol of chemical induction of long-term depression (cLTD), which consisted in application of *N*-methyl-d-aspartate (NMDA, 50 μM) for 20 min in Mg^2+^-containing KRB [28], indicating that a spontaneous or an LTP-like neuronal activity is required to induce CaN activation in astrocytes (Figure 3D). Most importantly, application of the cLTP protocol to purified astroglial cultures produced no effect on the localization of NY probe which remained concentrated to the cytosolic compartment with no significant increase in Nuc/Cyt NY ratio, indicating that the effect of cLTP on astroglial CaN activation required the presence of neurons to occur (Figure 3E). Stimuli which were used to induce nuclear NY translocation in astrocytes are summarized in Table 1.

Given that the cLTP protocol produced the most robust and fast translocation of NY in astrocytes, we further used it for detailed characterization of cLTP-like activity-induced astroglial CaN activation.

### 2.2. Neuronal Activity Induces Elevation of Cytosolic Calcium in Astrocytes

**Figure 4:**
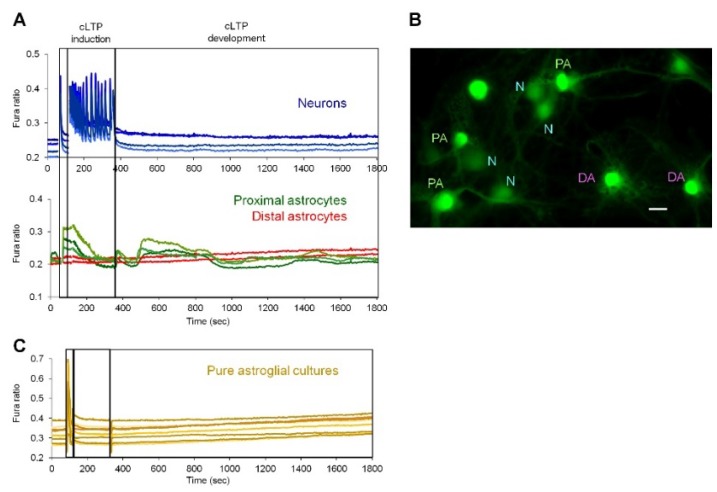
Delayed Ca^2+^ signals in proximal-to-neurons astrocytes induced by cLTP. (**A**) Ca^2+^ signals were detected by Fura-2 single cells imaging and analyzed separately in neurons (blue traces) and astrocytes (green and red traces). Note appearance of delayed Ca^2+^ signals in astrocytes which are in close apposition with neurons (green traces in panel **A** and “PA” (Proximal Astrocytes) label in **B**) but not in distal astrocytes, which are not in contact with neurons (“N”) (red traces in panel **A** and “DA” label in panel **B**). Representative traces and image are shown of cLTP induction experiments from 2–3 coverslips (technical replicates) for each of three independent culture preparations (biological replicates). Bar, 10 μm. **C**, Ca^2+^ signals were not detected in pure astroglial cultures stimulated with cLTP protocol.

For activation, CaN requires elevations of calcium concentrations in the cytosol. We investigated if the cLTP protocol induces calcium signals in astrocytes. Fura-2-loaded mixed hippocampal cultures were placed on the stage of epifluorescence setup and, after recording was started, KRB + Ca + Mg was changed first to Mg^2+^-free KRB + Ca solution to wash out Mg^2+^ ions. Then, cLTP cocktail was applied and, after 4 min, it was changed to KRB + Ca + Mg solution. After registration, Ca^2+^ signals were analyzed separately in neurons and astrocytes. As shown in Figure 4A, Mg^2+^-free KRB application induced a single Ca^2+^ spike, while cLTP cocktail induced a burst of Ca^2+^ spikes which lasted for the whole duration of the cLTP induction phase in neurons. After cLTP cocktail was washed-out, neurons either did not exhibit any Ca^2+^ activity (36.18%, *n* = 72), or generated single spikes (63.82%, *n* = 127) with a frequency of 0.32 ± 0.14 spikes/min. Example of an experiment without neuronal Ca^2+^ spikes in the cLTP development phase is shown in Figure 4, while in Figure S1 experiments are demonstrated in which neurons generated Ca^2+^ spikes. In astrocytes, robust Ca^2+^ signals were generated during the induction phase, which followed the neuronal burst of Ca^2+^ spikes and slightly decayed before removal of the cLTP cocktail. After the re-addition of KRB + Mg + Ca, the cultures were registered for longer time (25 min) and delayed Ca^2+^ transients were observed in a fraction of astrocytes. Closer examination of Fura-2 images revealed that only astrocytes which were juxtaposed to neuronal bodies or neuronal processed generated delayed Ca^2+^ signals (Figure 4B). These delayed astrocyte Ca^2+^ signals were not synchronous and could consist of more than one Ca^2+^ transient of different duration.

### 2.3. Activity-Induced Ca^2+^ Transients and CaN Activation in Astrocytes Depend on NMDA, mGluR5 and Store-Operated Ca^2+^ Entry

**Figure 5:**
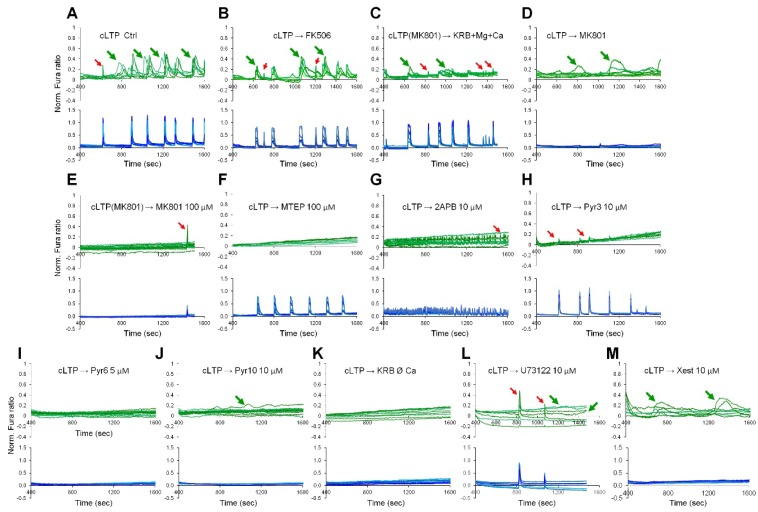
Pharmacological characterization of cLTP-induced astroglial Ca^2+^ signals. Fura-2 loaded hippocampal mixed neuron-astroglial cultures were stimulated with cLTP protocol and either not treated (**A**) or treated as indicated (unless otherwise stated cells were treated during cLTP development phase): FK506 (200 nM) (**B**); MK801 (100 μM, only in the phase of cLTP induction) (**C**); MK801 (100 μM, only in the phase of cLTP development) (**D**); MK801 (100 μM, during both phases, cLTP induction and development) (**E**); MTEP (100 μM) (**F**); 2APB (10 μM) (**G**); Pyr3 (10 μM) (**H**); Pyr6 (5 μM) (**I**); Pyr10 (10 μM) (**J**); Ø Ca^2+^ (**K**); U73122 (10 μM) (**L**); Xestospongin C (10 μM) (**M**). Ca^2+^ signals were analyzed separately in astrocytes (green traces) and in neurons (blue traces). Only parts of traces related to cLTP development phase are shown. cLTP induced Ca^2+^ elevation in astrocytes are indicated by green arrows. Artifacts related to neuronal Ca^2+^ spikes are indicated with red arrows. For corresponding full-length traces, see Figure S2.

**Figure 6:**
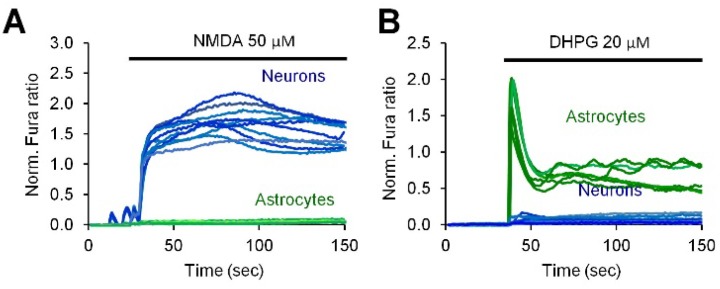
Cell type determination of NMDA and 3,5-Dihydroxyphenylglycine (DHPG) induced Ca^2+^ responses. Hippocampal mixed neuron-astroglial cultures were stimulated with 50 μM NMDA (**A**) or 20 μM DHPG (**B**). Ca^2+^ signals were detected using Fura-2 single cells imaging in astrocytes (green traces) and in neurons (blue traces).

Next, we used pharmacological inhibition to investigate the molecular mechanisms of neuronal activity-induced CaN activation in astrocytes. Ca^2+^ traces were registered on Fura-2 loaded cells in a separate set of experiments and the correlation of Ca^2+^ signals with CaN activation was achieved analyzing the responses of astrocytes to withdrawal of Ca^2+^ during the cLTP development phase or to pharmacological treatments. First of all, we investigated if application of FK506 would interfere with astroglial Ca^2+^ signals, and this was not the case since the Ca^2+^ signals in astrocytes during the development phase of cLTP could still be observed in presence of FK506 (Figure 5B). Further, we investigated the requirement of neuronal NMDA receptors. For this, MK801, a specific NMDA receptors inhibitor (50 μM), was applied either for only the phase of cLTP induction (Figure 5C) or only during the cLTP development phase after cLTP cocktail washout (Figure 5D) or during both the induction and the development phases (Figure 5E). When MK801 was applied for the entire duration of the experiment, Ca^2+^ activity was abrogated in both neurons and astrocytes, and no nuclear NY translocation was observed (Figure 5E). When MK801, instead, was applied solely during the phases of cLTP induction (Figure 5C) or cLTP development (Figure 5D and Table 2), neither astrocyte Ca^2+^ transients nor nuclear NY translocation were inhibited indicating that (1) astrocyte Ca^2+^ signals during the induction phase were secondary to neuronal Ca^2+^ signals; and (2) neuronal Ca^2+^ signals during the induction phase are necessary for the astroglial Ca^2+^ signals to occur during the phase of cLTP development. Next, involvement of the metabotropic glutamate receptor, mGluR5, was investigated. Figure 5F and Table 2 show that application of MTEP, a specific mGluR5 antagonist (100 μM), during the cLTP development phase abolished both astroglial Ca^2+^ signals and NY nuclear translocation. This result suggests the requirement of mGluR5 for the activity-induced CaN activation, although, in the current setting, it does not discriminate between neuronal and astroglial localization of mGluR5. Previously, we published that in rat hippocampal mixed neuron-astroglial culture neurons preferentially responded to NMDA while astrocytes responded to 3,5-Dihydroxyphenylglycine (DHPG) [29], a powerful mGluR agonist. We, therefore, stimulated our mixed cultures either with NMDA (50 μM) or DHPG (20 μM). Expectedly, only neurons responded to NMDA stimulation (Figure 6A) while only astrocytes responded to DHPG (Figure 6B), providing thus an indirect evidence that NMDA inhibitor MK801 acted on neuronal NMDA receptors, while MTEP inhibited astroglial mGluR5.

Store-operated Ca^2+^ entry has been shown to mediate long lasting Ca^2+^ elevations in cultured astrocytes [30]. We, therefore, used a panel of drugs known to inhibit SOCE (Table 2) albeit by different mechanisms. Three of them, namely, 2APB (50 μM, a non-specific transient receptor potential (TRP) receptor and Orai1 inhibitor, that inhibits also InsP3 receptors [31] (Figure 5G), Pyr3 (10 μM, inhibitor of Orai1 and TRPC3) (Figure 5H) and Pyr6 (5 μM, specific Orai1-mediated SOCE inhibitor) [32] (Figure 5I), when applied after washout of the cLTP cocktail, efficiently inhibited both Ca^2+^ signals and CaN activation in astrocytes. However, Pyr10 (10 μM, specific TRPC3-mediated SOCE inhibitor [32]) (Figure 5J), failed to inhibit Ca^2+^ elevations, thereby ruling out TRPC3 involvement in astrocyte activation by neuronal activity. Note that 2APB somewhat strongly augmented frequency of neuronal Ca^2+^ spikes during the cLTP development phase (Figure S2G) resulting in the appearance of numerous artefacts in neighboring astrocytes (Figure 5E). The requirement of extracellular Ca^2+^ was also confirmed by withdrawal of Ca^2+^ from the extracellular buffer (Figure 5K). Of note, we could not detect any discernible ER-dependent Ca^2+^ release in the absence of extracellular Ca^2+^, as recently shown and discussed in [33]. We also attempted to investigate the involvement of astroglial phospholipase C (PLC), which synthetize InsP3, and InsP3 receptors (InsP3Rs) by exploiting specific cell permeant inhibitors. However, U73122 (10 μM; Figure 5L) and xestospongin C (10 μM; Figure 5M), which, respectively, target PLC and InsP3Rs, failed to completely inhibit either astroglial Ca^2+^ signals or CaN activation. These last experiments, however, may not be conclusive as both U73122 and xestospongin C may require up to 10 min to efficiently inhibit InsP3 production and InsP3Rs, respectively. Unfortunately, their addition before the phase of cLTP induction would have compromised InsP3R-mediated signaling in neurons. In Figure 5, the traces are shown separately for astrocytes and neurons and are limited to the cLTP development phase. Full length Ca^2+^ traces are provided in Supplementary Figure S2.

## 3. Discussion

In this report, we provide an in vitro proof of principle of activation of astroglial CaN by neuronal activation. The main findings are: (1) in mixed neuron-astroglial hippocampal primary cultures, cLTP induction protocol, which specifically stimulates neuronal activity, induced intracellular Ca^2+^ signals and robust CaN activation in astrocytes, and (2) astroglial Ca^2+^ signals and CaN activation required extracellular Ca^2+^ entry via the SOCE mechanism. 

Although, to our knowledge, there is no data published to date that neuronal activity may result in CaN activation in astrocytes, it has been reported that the increase in neuronal activity is able to induce CaN activation and nuclear translocation of NFAT in pericytes in cortical slices [34]. This landmark contribution suggests that neuronal activity may, in fact, activate CaN in non-neuronal cells. Now, we demonstrate that, in an in vitro setting, CaN may be activated also in astrocytes.

Calcineurin is activated by a specific pattern of calcium signaling which is characterized by low and sustained (minutes) elevations of baseline cytosolic calcium levels [35], while the specificity of downstream CaN targets activation can be further achieved by a specific temporal pattern of Ca^2+^ elevations [36]. Stimulation of neuronal activity is known to produce calcium signals in astrocytes in vivo [37,38,39,40,41,42,43,44,45], including awake animals [46,47], in brain slice preparations [34,48,49,50,51] and in mixed neuron-astrocyte primary cultures [52,53]. Most of these signals have been registered as short single or oscillatory transients with duration from several milliseconds to seconds [38,39,44]. Some of them, however, lasted long enough (tens of seconds to minutes) [41,54] to speculate that they would be sufficient for CaN activation. Recent experiments employing fast 3D calcium imaging suggest that the spatio-temporal pattern of Ca^2+^ signals in astrocytes is extremely complex, and depends on the nature of Ca^2+^-related receptors/channels expressed in a particular subdomain of astroglial plasma membrane [55]. Accordingly, it can be speculated that localized CaN activation may be necessary to achieve spatial specificity of processes controlled by the astrocytes. Further experiments are needed to demonstrate and characterize in vivo activity-dependent CaN activation in astrocytes.

Our findings suggest that neuronal activity induces Ca^2+^ entry in astrocytes via SOCE mechanism. SOCE is one of the fundamental mechanisms of astroglial Ca^2+^ signalling [56] and is involved in the generation of Ca^2+^ oscillations, refilling of the endoplasmic reticulum with Ca^2+^ [57], astroglial cytokine production [58] and astroglial metabolism [59]. Spontaneous Ca^2+^ oscillations in vivo in fine astroglial processes were shown to involve Ca^2+^ entry through the plasma membrane, probably through store-operated channels [60,61]. Concerning pathological conditions, SOCE is involved in the invasion of human glioblastoma [62], and is augmented in primary [30] astrocytes from an AD mouse model. Our results provide the physiological rationale for SOCE activation in astrocytes by neuronal activity. We also show that ionotropic NMDA and metabotropic mGluR5 receptors are involved in SOCE generation by LTP-like neuronal activity. Although it was impossible to discriminate the cell type on which NMDA or mGluR5 reside in the current setting, our previous observations [29] and direct stimulation of mixed cultures either with NMDA or DHPG (Figure 6), suggest that NMDAR are expressed in neurons while mGluR5 is located in astrocytes. Furthermore, cultured astrocytes are somewhat more sensitive than neurons to DHPG, as 20 μM DHPG is enough to induce Ca^2+^ increase in astrocytes, but not in neurons [29] (Figure 6), while 200 μM DHPG were used to elicit mGluR5-dependent Ca^2+^ transients in neurons [63]. Last, astroglial mGluR5 receptors are required to sustain long lasting Ca^2+^ entry in cultures astrocytes that was efficiently blocked by SOCE inhibitors [30].

SOCE is known to activate CaN/NFAT axis and modulate gene transcription in a number of cell types, including T-lymphocytes and mast cells [64], cardiomyocytes [65], skeletal muscle cells [66] and in neural progenitor cells [67]. We show now that SOCE is also required for neuronal activity-induced CaN activation in astrocytes in vitro while future experiments will show if SOCE activates CaN in astrocytes also in intact brain. Regarding the nature of SOCE channels, our pharmacological survey suggests that Orai1-containing channels are operative in astrocytes (efficiently inhibited by Orai1-blocking Pyr3 and Pyr6, [32]), while participation of TRPC3 is questionable, since Pyr10, a specific TRPC3 inhibitor [32]), was not as efficient as other SOCE blockers. In line with this, in our previous report, Pyr10 failed to inhibit the DHPG-induced after-peak Ca^2+^ entry, while it was efficiently inhibited by Pyr6 [30]).

In neurons, CaN is involved in long-term changes during neuronal plasticity, e.g., forebrain neuronal deletion of CaN specifically affects bidirectional synaptic plasticity and episodic-like working memory [18], and inactivation of CaN is essential for the onset of transcriptional remodeling during long-term plasticity and memory formation [17,19]. Our results suggest that also in astrocytes activity-induced CaN activation may be involved in long-term transcriptional remodeling leading to structural, biochemical and functional astroglial plasticity [68,69,70]. Further studies are necessary to confirm this hypothesis. 

The present report is a proof of principle in vitro study, and is not devoid of limitations, the two principal of which are: (1) the in vitro setup, which, obviously, only in part replicates the complex LTP phenomenon occurring in intact brain or even in brain slices; and (2) chemical instead of electrical LTP induction. We have consciously accepted these limitations for the following reasons. The in vitro setting proved to be simple and highly reproducible both in terms of lentiviral NY infection and in terms of cLTP and NY nuclear translocation. Thereby, it allowed us to rule out astrocytes as a primary target of cLTP protocol. Yet, we used hippocampal primary cultures and the effect may be different in cultures prepared from the other brain regions. Regarding cLTP induction, there are several protocols which are basically the modifications of two principal variants: the first consists in the application of a cocktail containing protein kinase A (PKA) activators, like forskolin and rolipram [28,71,72], which recruits downstream targets of PKA-dependent phosphorylation involved in LTP induction. For obvious reasons, this protocol is not specific to neurons and would induce PKA-dependent phosphorylation also in astrocytes. The second variant consists in the relieve of GABA-dependent inhibition by blocking ionotropic GABA(A) receptors with bicuculline and strychnine and in facilitation of NMDA receptors with glycine and Mg^2+^-free buffer [73,74,75,76]. We have chosen this second protocol because it minimally interferes with astrocyte biochemistry and does not result in NY translocation in pure astroglial cultures. Relieve of GABA-dependent inhibition alone is known to increase neuronal firing rate [77] and, notably, it was sufficient to induce nuclear translocation of NY and, hence, CaN activation, although significantly later than it was achieved by cLTP. Similar effect was achieved by Mg^2+^ withdrawal, which indicates that different strategies to facilitate neuronal activity may lead to CaN activation in astrocytes, although with different temporal pattern. 

An important question which remains unanswered in this work is the nature of the mediator which is released by neurons to induce the astroglial response. Assuming that astrocytic mGluR5 receptors are involved in astrocyte activation, it is plausible to speculate that the messenger is glutamate released during neuronal activity. This has been demonstrated in vitro [78] and in situ [79]. However, in vivo it has been shown that mGluR5 is downregulated during postnatal development and no longer active in adult astrocytes [80]. Therefore, the mechanisms of the activity-induced generation of Ca^2+^ signals in astrocytes, as well as of CaN activation, may differ between ex vivo and in vivo preparations, and may also depend on age at which the preparation is made.

**Figure 7:**
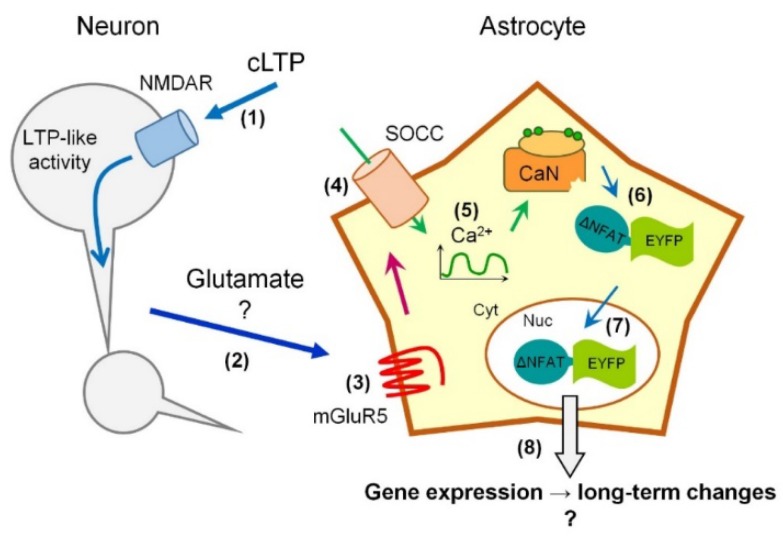
Model of LTP-like neuronal activity-induced CaN activation in astrocytes. LTP-like neuronal activity, possibly through activation of NMDA receptors (**1**) and glutamate release form neuronal terminals (**2**), induces Ca^2+^ entry through the plasma membrane of adjacent astrocytes, possibly implicating astrocytic mGluR5 receptors (**3**). The Ca^2+^ entry occurs through Orai1-containing SOCE channels (SOCC) (**4**) resulting in delayed long-lasting Ca^2+^ transients (**5**) and activation of CaN inside the astrocytes (**6**), which, in turn, leads to nuclear translocation of CaN sensor NY (ΔNFAT-EYFP) (**7**). The latter event is quantified as an increase in ratio between NY fluorescence in the nucleus (Nuc) and the cytoplasm (Cyt). Increase in CaN activity in astrocytes may lead to transcriptional remodeling and long-term changes (**8**). “?“ in (**2**) and (**8**) indicates questionable points.

In summary, we propose a model (Figure 7) in which LTP-like neuronal activity, possibly through activation of NMDA receptors and glutamate release form neuronal terminals, induces Ca^2+^ entry through the plasma membrane, possibly implicating astrocytic mGluR5 receptors. The Ca^2+^ entry occurs through Orai1-containing SOCE channels and results in activation of CaN inside the astrocytes, which, in turn, leads to activation and nuclear translocation of CaN sensor NY. CaN activation in astrocytes may lead to transcriptional remodeling and long-term changes analogous to what occur during neuronal plasticity. In this model, we provide a framework for future investigation of astroglial CaN activation during neuronal activity and plasticity in physiology and pathology of CNS.

## 4. Materials and Methods 

### 4.1. Animals

C57Bl/6 mice have been purchased from Charles River Laboratories (Calco (Lecco), Italy). The animals have been given food and water ad libitum; light/dark cycle has been automatically controlled in respect with the natural circadian rhythm and the temperature has been thermostatically regulated. Animals were managed in accordance with European directive 2010/63/UE and with Italian low D.l. 26/2014. The procedures were approved by the local animal-health and ethical committee (Università del Piemonte Orientale) and were authorized by the national authority (Istituto Superiore di Sanità; authorization number N. 22/2013, 3 February 2013). 

### 4.2. Primary Hippocampal Mixed and Astroglial Cultures

To prepare mixed neuron-astroglial hippocampal primary cultures, new-born mice (less than one day old) were used. Pups were sacrificed by decapitation and hippocampi were dissected in cold HBSS (Sigma, Darmstadt, Germany, Cat. H6648). After dissection, hippocampi were digested in 0.1% Trypsin (Sigma, Cat. T4049) for 20 min in 37 °C water bath. Then, trypsin was neutralized by addition of Dulbecco’s Modified Eagle’s Medium (DMEM, Sigma, Cat. D5671) supplemented with 10% heat-inactivated fetal bovine serum (FBS, Life Technologies, Monza, Italy, Cat. 10270-106) and tissue was spun at 300× *g* for 5 min. Tissue pellet was resuspended in HBSS supplemented with 10% FBS and dissociated by 30 strokes of a 1000 μL automatic pipette. After pipetting, the tissues were left for 5 min to allow sedimentation of un-dissociated tissue, and cell suspension was transferred to a new tube and centrifuged at 250× *g* for 5 min. Hippocampal cells were resuspended in Neurobasal-A medium supplemented with 2% B-27 and with 2 mg/mL glutamine, 10 U/mL penicillin and 100 µg/mL streptomycin, and plated onto Poly-l-lysine-coated coverslips (0.1 mg/mL). For NY translocation time-course, the cells were plated onto 13 mm round coverslips in 24 well plates, 2 × 10^4^ cells per well. For Fura-2 imaging, the cells were spotted (2 × 10^4^ cells per spot) on 24 mm round coverslips in 6 well plates. Half of medium was changed every 5 days. Cultures were used for treatments and experiments after 12 days in vitro (DIV12). At DIV12-DIV14 the neuron/astrocyte ratio was 1.29 ± 0.49 as was counted in 34 coverslips from at least 8 independent cultures.

To prepare purified astroglial cultures, one to three-day old pups were sacrificed by decapitation, hippocampi were rapidly dissected and placed in cold HBSS. Tissue was digested in 0.25% trypsin (37 °C, 20 min), washed in complete culture medium ((DMEM), supplemented with 10% FBS, and with 2 mg/mL glutamine, 10 U/mL penicillin and 100 µg/mL streptomycin (all from Sigma) and resuspended in cold HBSS supplemented with 10% FBS. After 30 strokes of dissociation with an automatic pipette, cell suspension was centrifuged (250× *g*, 5 min), pellet was resuspended in complete medium and plated in 100 mm Falcon culture dishes, pretreated with 0.1 mg/mL Poly-l-lysine (Sigma). At sub-confluence (DIV5-10), cells were detached with trypsin and microglial cells were removed by magnetic-activated cell sorting (MACS) negative selection using anti-CD11b conjugated beads and MS magnetic columns (Miltenyi Biotech, Bologna, Italy, Cat. 130-093-634). After MACS, astrocytes were counted and plated for experiments as described above. Virtually no microglial cells have been detected in purified astroglial cultures after MACS procedure as was assessed by immunostaining with anti-Iba1 antibody (1:500, D.B.A., Segrate, Italy, Cat. 019-19741) [81].

### 4.3. Lentivirus Production and Transduction

mCherry-H2Bc-ΔNFAT-EYFP-expressing pFUW (pFUW-NY) third generation lentiviral vector was a kind gift from Prof. Alexander Flügel, Göttingen [23]. The production of infectious lentiviral particles was done using polyethylene glycol (PEG) method as describe elsewhere [82]. Briefly, HEK293T cells were transfected with four plasmids (2.75 μg pMDLg.pRRE, 1 μg pRSV.Rev, 1.5 μg pMD2.VSVG, and 6 μg pFUW-NY per 100 mm dish). After 48–72 h medium was collected and viral particles were precipitated by adding 1/5 of 5× PEG solution (200 g PEG (Sigma, Cat. n. 89510), 12 g NaCl, 1 mL Tris 1 M, pH 7.5, in ddH_2_O to a final volume of 500 mL) overnight. On the next day the precipitate was concentrated by centrifugation (2800× *g* at 4 °C for 30 min) and resuspended in HBSS (1/100 of the original medium volume, aliquoted and stored at −80 °C. All manipulations with lentiviral vector were conducted in biosafety level 2 environment in accordance with Italian ministry of health-approved protocol.

DIV10-12 mixed neuron-astroglial cultures or 50% confluent purified astrocytes were transduced by adding (50 μL/mL) concentrated NY-expressing viral particles. Experiments were conducted 2–4 days after infection. For each condition and time-point, at least 2 coverslips were used (technical replicates) in three independent culture preparations (biological replicates).

### 4.4. Induction of cLTP and NY Translocation Quantification

cLTP was induced by a cLTP cocktail containing 20 μM bicuculline, 1 μM strychnine, 200 μM glycine in Mg^2+^-free Krebs-Ringer buffer (KRB, 135 mM NaCl, 5 mM KCl, 0.4 mM KH_2_PO_4_, 5.5 mM glucose, 20 mM HEPES, pH 7.4). First the cells were rinsed by Mg^2+^-free KRB along to wash out Mg^2+^ ions. Then, cLTP cocktail was applied for 4 min to induce LTP (cLTP induction phase). After this, the cLTP cocktail was changed to Mg^2+^- and Ca^2+^-containing KRB (KRB + Mg + Ca) and kept until the cells were fixed or imaged (cLTP development phase). At indicated time-points after cLTP induction, KRB + Mg + Ca was quickly removed and cells were fixed with 4% formalin in PBS (20 min, room temperature (RT)). Fixed cells were washed 3 times with PBS and mounted on microscope slides using SlowFade^®^ Gold Antifade mountant (Life Technologies, Monza, Italy).

Fixed cells were imaged on a Leica DMI6000 epifluorescent microscope equipped with Polychrome V monochromator (Till Photonics, Graefelfing, Germany) and S Fluor ×40/1.3 objective (Leica, Buccinasco, Italy). The cells were alternatively excited by 488 and 546 nm and emission light was filtered through 520/20 and 600/40 nm bandpass filters, respectively, and collected by a cooled CCD camera (Leica, Hamamatsu, Japan). The fluorescence signals were acquired and processed using MetaMorph software (Molecular Device, Sunnyvale, CA, USA). 

To quantify NY translocation, 5 random fields were photographed from each coverslip, and the astrocytes with clear expression of NY sensor were used for analysis (3–15 astrocytes per field). For each cell two regions of interest (ROIs) were placed inside the nucleus (which was evidenced by mCherry-H2Bc expression) and two in different sides of the cytosol close to the nucleus. For each cell, the fluorescence intensity of two nuclear (Nuc) and two cytosolic (Cyt) ROIs, respectively, measured in green channel, was averaged and Nuc/Cyt ratio was obtained by dividing the resulting Nuc fluorescence to Cyt fluorescence. The data are expressed as mean ± SEM for each cell analyzed form three independent culture preparations (e.g., Figure 3).

### 4.5. Induction of cLTP and NY Translocation Quantification

For Fura-2 Ca^2+^ imaging experiments, the cells were first loaded with 2 μM Fura-2-AM in presence of 0.02% of Pluronic-127 (both from Life Technologies) and 10 μM sulfinpyrazone (Sigma) for 20 min at RT. Fura-2 loaded cells were washed in KRB + Mg + Ca and allowed to de-esterify for 20 min before cLTP induction. 

After that, the coverslips were mounted into acquisition chamber and placed on the stage of a Leica DMI6000 epifluorescence microscope equipped with S Fluor ×40/1.3 objective. The probe was excited by alternate 340 and 380 nm using a Polychrome IV monochromator and the Fura-2 emission light was filtered through 520/20 bandpass filter and collected by a cooled CCD camera (Hamamatsu, Japan). The fluorescence signals were acquired and processed using MetaFluor software (Molecular Device, Sunnyvale, CA, USA). To quantify the differences in the amplitudes of Ca^2+^ transients the ratio values were normalized using the formula ΔF/F0 (referred to as normalized Fura-2 ratio, “Norm. Fura ratio”). At least two coverslips for each of three independent culture preparation were imaged for each condition. In mixed neuron-astroglial cultures, astrocytes were recognized as flat polygonal or star-like cells, while neurons were recognized by round bodies with few processes located on upper focal plane above the astrocytes. The cells with uncertain morphology were not taken in consideration.

### 4.6. Pharmacological Reagents

MK801 (Cat. 0924, stock 100 mM in DMSO), MTEP (Cat. 2921, stock 100 mM in DMSO), 2APB (Cat. 1224, stock 50 mM in DMSO), Xestospongin C (Cat. 1280, stock 2 mM in DMSO), U73122 (Cat. 1268, stock 10 mM in DMSO), NMDA (Cat. 0114, stock 100 mM in H_2_O) and DHPG (Cat. 0342, stock 10 mM in PBS) were from Tocris (Bristol, UK). Pyr3 (Cat. P0032, stock 10 mM in DMSO), Pyr6 (Cat. SML1241, stock 10 mM in DMSO) and Pyr10 (Cat. SML1243, stock 10 mM in DMSO) were from Sigma.

### 4.7. Statistical Analysis 

Statistical analysis was performed using GraphPad Prism software v.7. For analysis of Nuc/Cyt NY ratio (Figure 3) each dataset at indicated time-points was compared with respective control using a two-tailed unpaired Students’s *t*-test. Differences were considered significant at *p* < 0.05. Data are expressed as mean ± SEM.

## Figures and Tables

**Table 1:** Stimuli which induced nuclear NY translocation in astrocytes.

Drug/Condition	Astroglial Ca^2+^ Elevation	NY Nuclear Translocation
cLTP	Yes Ca^2+^ signals	Translocation after 10 min
LTD	NA	No translocation
Bicuculline alone 40 μM	NA	Translocation after 1 h
Ø Mg^2+^	NA	Translocation after 2 h
cLTP on pure astrocytes	No Ca^2+^ signals	No translocation

Unless otherwise stated, the condition is applied to mixed neuron-astroglial cultures. NA, not assayed.

**Table 2:** Pharmacological characterization of cLTP-induced astroglial Ca^2+^ signals and NY translocation.

Pharmacological Treatment	Astroglial Ca^2+^ Elevation	NY Nuclear Translocation
cLTP → KRB + Mg + Ca	Yes	Yes
cLTP → Ø Ca^2+^	No	No
cLTP → KRB + Mg + Ca + FK506	Yes	No
cLTP → KRB + Mg + Ca + cyclosporine A	Yes	No
cLTP (MK801) → KRB + Mg + Ca	Yes	Yes
cLTP → KRB + Mg + Ca + MK801	Yes	Yes
cLTP (MK801) → KRB + Mg + Ca + MK801	No	No
cLTP → KRB + Mg + Ca + MTEP	No	No
cLTP → KRB + Mg + Ca + 2APB	No	No
cLTP → KRB + Mg + Ca + Pyr3	No	No
cLTP → KRB + Mg + Ca + Pyr6	No	No
cLTP → KRB + Mg + Ca + Pyr10	Yes	Yes
cLTP → KRB + Mg + Ca + Xestospongin	Yes	Yes
cLTP → KRB + Mg + Ca + U73122	Yes	Yes

## Supplementary Materials

Supplementary materials can be found at http://www.mdpi.com/1422-0067/19/10/2997/s1.

## Abbreviations

CaN	Calcineurin
cLTP	Chemical Long-Term Potentiation
cLTD	Chemical Long-Term Depression
KRB	Modified krebs-Ringer Buffer
MK801	(5*S*,10*R*)-(+)-5-Methyl-10,11-dihydro-5*H*-dibenzo[a,d]cyclohepten-5,10-imine Maleate
MTEP	3-((2-Methyl-1,3-thiazol-4-yl) ethynyl) Pyridine Hydrochloride
DHPG	(*RS*)-3,5-Dihydroxyphenylglycine
U73122	1-[6-[[(17β)-3-Methoxyestra-1,3,5(10)-trien-17-yl]amino]hexyl]-1*H*-pyrrole-2,5-dione
NMDA	*N*-Methyl-d-aspartic Acid
TRPC3	Transient Receptor Potential C3
2APB	2-Aminoethoxydiphenylborane
Pyr3	1-[4-[(2,3,3-Trichloro-1-oxo-2-propen-1-yl)amino]phenyl]-5-(trifluoromethyl)-1*H*-pyrazole-4-carboxylic Acid
Pyr6	*N*-[4-[3,5-Bis(trifluoromethyl)-1*H*-pyrazol-1-yl]phenyl]-3-fluoro-4-pyridinecarboxamide
Pyr10	*N*-[4-[3,5-Bis(trifluoromethyl)-1*H*-pyrazol-1-yl]phenyl]-4-methyl-benzenesulfonamide
DMSO	Dimethyl Sulfoxide
SOCE	Store-Operated Calcium Entry
