# Supplementary material for: Neuronal Activity-Dependent Activation of Astroglial Calcineurin in Mouse Primary Hippocampal Cultures
Source: Int J Mol Sci. 2018 Sep 30;19(10):2997. doi: 10.3390/ijms19102997 (PMC6213389; doi:10.3390/ijms19102997)
Supplement: Supplementary file 1 [file ijms-19-02997-s001.zip › supplementary/Suppl Figures v.2.pdf]

Supplementary Material

Neuronal activity-dependent activation of astroglial calcineurin in mouse primary hippocampal cultures

Dmitry Lim, Lisa Mapelli, Pier Luigi Canonico, Francesco Moccia and Armando A Genazzani

Supplementary material	Title	File
Supplementary video 1	Ionomycin-induced nuclear translocation of NY calcineurin sensor	Suppl_Video_1
Supplementary Figure 1	Examples of neuronal and astroglial Ca^{2+} signals induced by cLTP induction protocol	Suppl_Figures
Supplementary Figure 2	Neuronal Ca^{2+} transients registered during stimulation of mixed neuron-astroglial hippocampal cultures with cLTP protocol.	Suppl_Figures

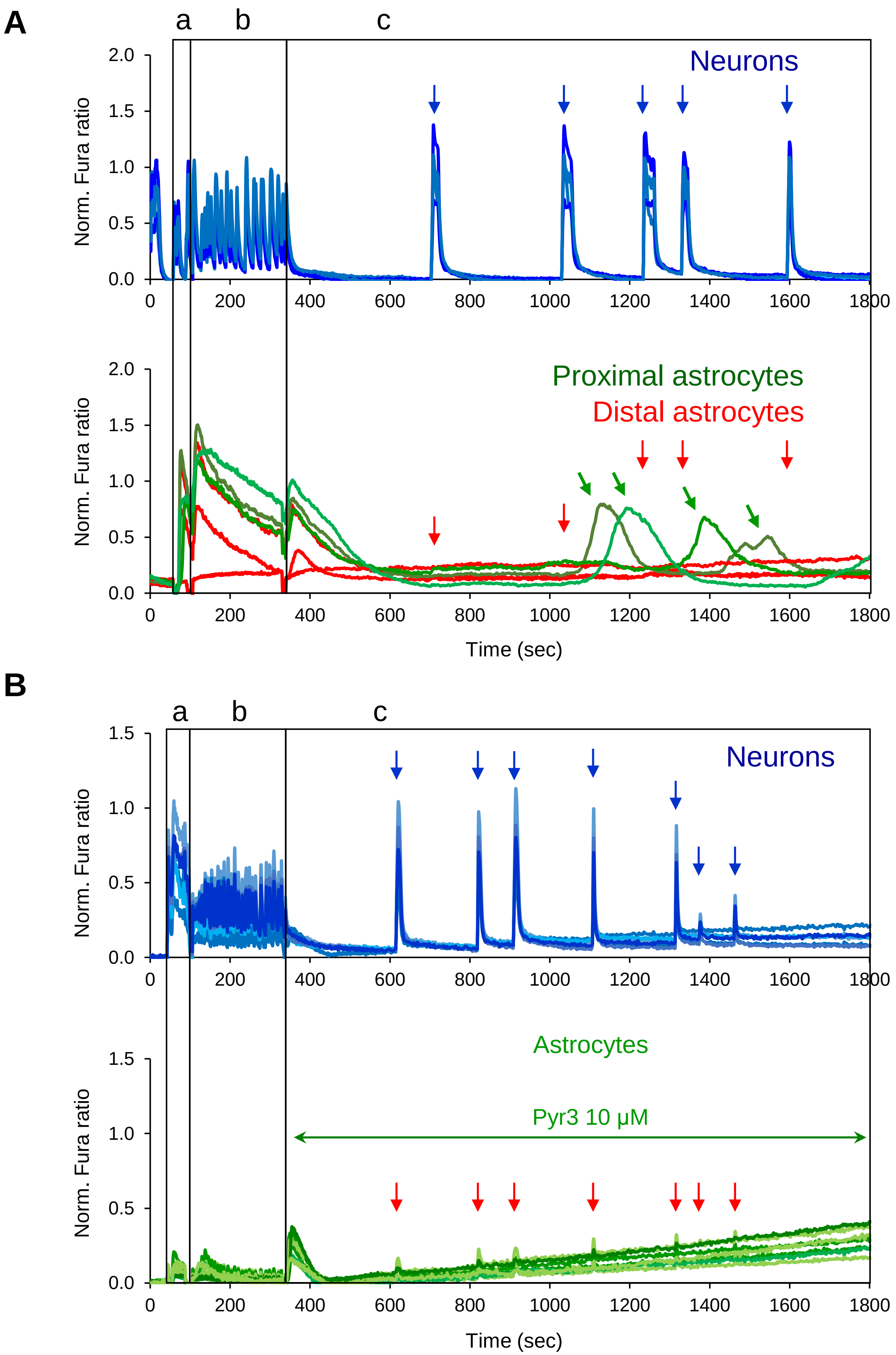

A
a
b
c
Neurons
Norm. Fura ratio
Proximal astrocytes
Distal astrocytes
Time (sec)
B
a
b
c
Neurons
Norm. Fura ratio
Astrocytes
Pyr3 10 μM
Time (sec)

Supplementary Figure 1. Representative Fura-2 Ca^{2+} imaging experiments demonstrating cLTP-induced Ca^{2+} transient in neurons (blue traces) and astrocytes (in juxtaposition to neurons, green traces, or distal astrocytes, red traces). In black frame, the sector (a) indicates Mg^{2+} washout; sector (b), cLTP induction phase; and sector (c), cLTP development phase. During the cLTP development phase, blue arrows indicate Ca^{2+} spikes in neurons, green arrows – delayed cLTP-induced Ca^{2+} transients in astrocytes, and red arrows – artifacts due to light interference with neuronal Ca^{2+} spikes. A, control induction of cLTP; B, inhibition of cLTP-induced delayed astroglial Ca^{2+} transients with SOCE inhibitor Pyr3

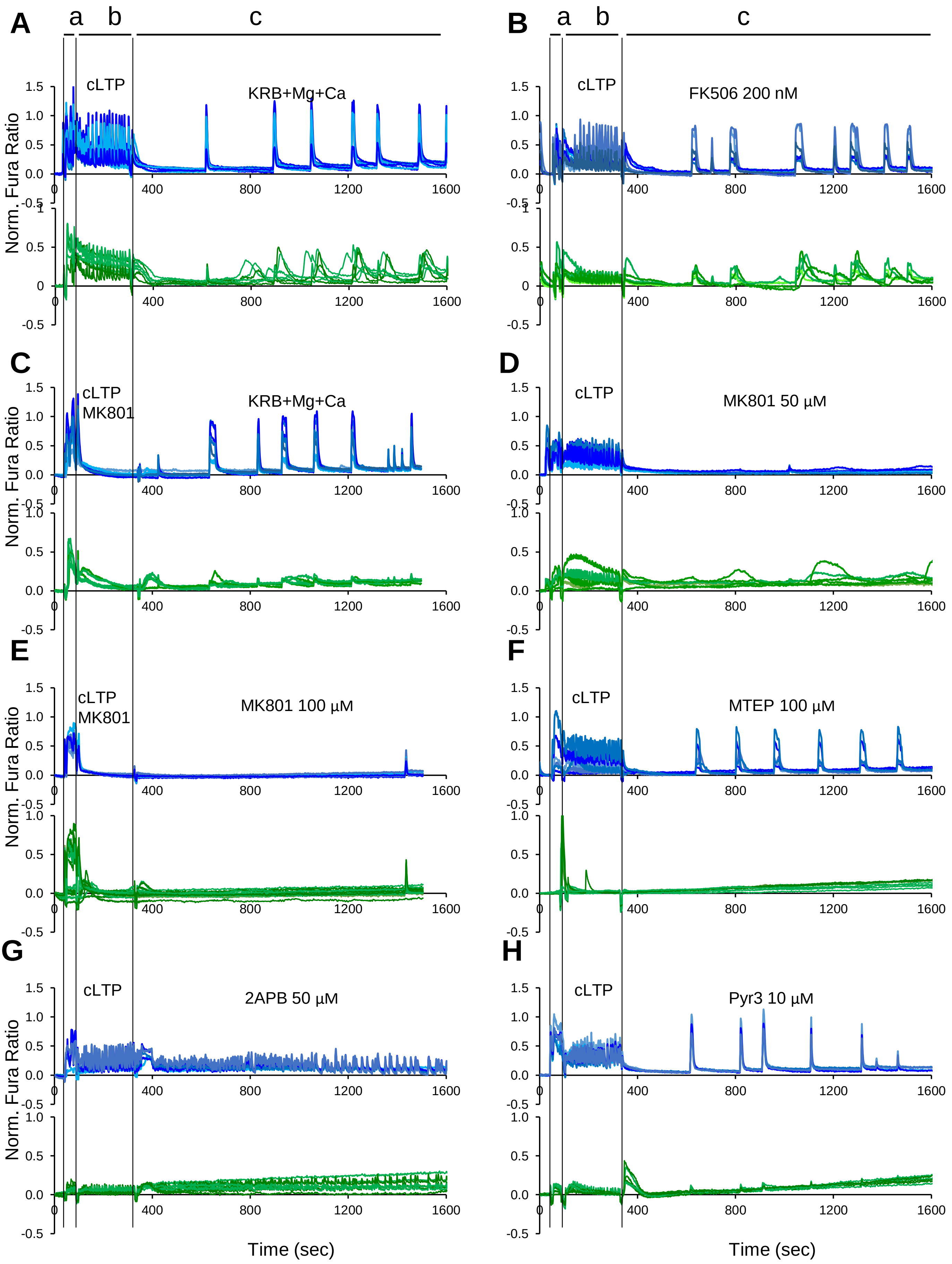

A
B
C
D
E
F
G
H
a
b
c
cLTP
KRB+Mg+Ca
FK506 200 nM
cLTP MK801
MK801 50 μM
MK801 100 μM
MTEP 100 μM
2APB 50 μM
Pyr3 10 μM
Norm. Fura Ratio
Time (sec)

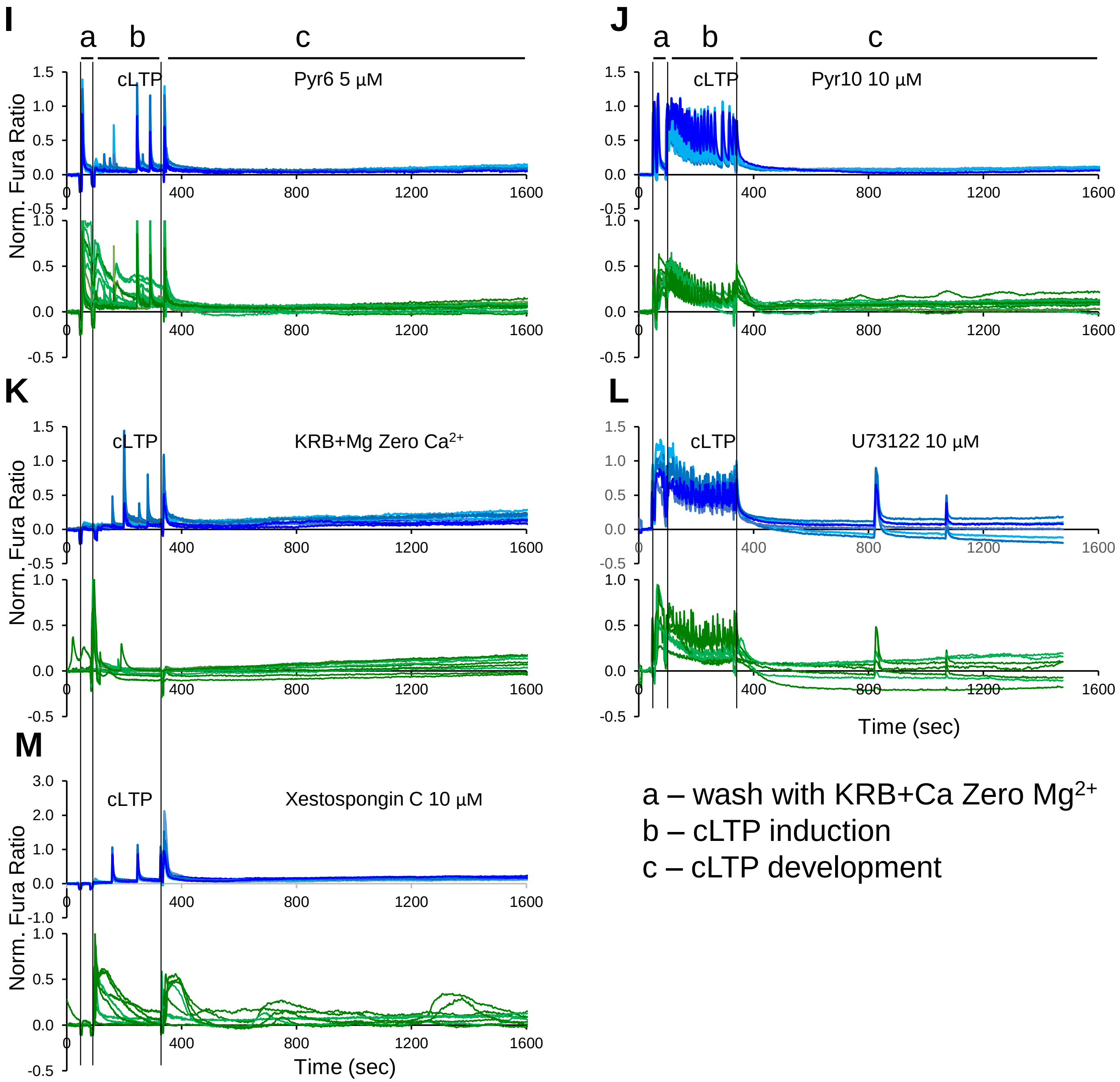

Supplementary Figure 2. Neuronal (blue) and astroglial (green) Ca^{2+} transients registered during stimulation of mixed neuron-astroglial hippocampal cultures with cLTP protocol. Panel labels correspond to panels in Figure 5 described in the main text. Sector (a) indicates Mg^{2+} washout with KRB+Ca Zero Mg^{2+}; sector (b), cLTP induction phase; and sector (c), cLTP development phase. In the sector (b), cLTP indicates the cLTP induction phase.
